# Descriptive Pathological Study of Avian Schistosomes Infection in Whooper Swans (*Cygnus cygnus*) in Japan

**DOI:** 10.3390/ani10122361

**Published:** 2020-12-10

**Authors:** Mohamed S. Ahmed, Reda E. Khalafalla, Ashraf Al-Brakati, Tokuma Yanai, Ehab Kotb Elmahallawy

**Affiliations:** 1 Department of Pathology, Faculty of Veterinary Medicine, Kafrelsheikh University, Kafrelsheikh 33516, Egypt; aosayedahmed@yahoo.com; 2 Department of Veterinary Pathology, Gifu University, 1-1 Yanagido, Gifu 501-1193, Japan; 3 Department of Parasitology, Faculty of Veterinary Medicine, Kafrelsheikh University, Kafrelsheikh 33516, Egypt; redabast@hotmail.de; 4 Department of Human Anatomy, College of Medicine, Taif University, P.O. Box 11099, Taif 21944, Saudi Arabia; a.albrakati@tu.edu.sa; 5 Laboratory of Wildlife and Forensic Pathology, Biomedical Science Examination and Research Center, Department of Veterinary Medicine, Faculty of Veterinary Medicine, Okayama University of Science, Okayama 700-0005, Japan; tokumayanai@gmail.com; 6 Department of Zoonoses, Faculty of Veterinary Medicine, Sohag University, Sohag 82524, Egypt

**Keywords:** schistosome, *Allobilharzia visceralis*, whooper swans, obstructive phlebitis

## Abstract

**Simple Summary:**

Avian schistosomes are a group of parasites responsible for most of the reported cases of cercarial dermatitis outbreaks. Among others, *Trichobilharzia* is considered the largest genus of avian Schistosomatidae, and it infects more than 40 avian species. The present study involves a descriptive pathological study of avian schistosome in 54 whooper swans (*Cygnus cygnus*) from various rescue/rehabilitation centers in Honshu, Japan. Interestingly, adult schistosomes were detected in the lumen of mesenteric, serosal, portal, and testicular veins, in the capillaries of the intestinal lamina propria, and in the sinusoids of the adrenal gland, spleen, and liver of 23 (42.59%) swans. Schistosomes were assumed to be *Allobilharzia visceralis* based on the morphological characteristics of the worm and eggs found at histopathological examination of internal organs, along with suggestive pathological findings as well as the pathological findings. Collectively, the present study provides novel descriptive pathological data about schistosome infection in whooper swans with new insights on their role in the transmission and spreading of avian schistosomes in Japan.

**Abstract:**

Cercarial dermatitis, or Swimmer’s itch, is one of the emerging diseases caused by the cercariae of water-borne schistosomes, mainly *Trichobilharzia* spp. Since the zoonotic potential of *Allobilharzia visceralis* is still unknown, studies on this schistosome would be helpful to add knowledge on its possible role in causing human infections. In the present study, 54 whooper swans (*Cygnus cygnus*) from rescue/rehabilitation centers in Honshu, Japan, were necropsied to identify the cause of death. Grossly, 33 (61.11%) swans were severely emaciated and 23 (42.59%) had multiple reddened areas throughout the length of the intestine with no worms detected in the internal organs. Microscopically, adult schistosomes were found in the lumen of the mesenteric, serosal, portal, and testicular veins, in the capillaries of the intestinal lamina propria, and in the sinusoids of the adrenal gland, spleen, and liver of 23 (42.59%) swans. Hypertrophy of veins containing adult worms was identified in 15 (27.77%) swans, and vascular lumen obliteration was observed in 8 (14.81%) swans. Mild to severe villous atrophy and superficial enteritis were observed in 8 birds (14.81%), whereas bile pigments and hemosiderin were detected in the livers of 14 (25.92%) and 18 (33.33%) swans, respectively. In three swans (5.55%), schistosome parasites were found in the subcapsular veins of the testes. The schistosomes in the present study were assumed to be *A. visceralis* based on the microscopical and histological evidence of adult schistosomes found in the lumen of veins as well as the infection pathology, which was very similar to the schistosome-induced pathology previously reported in swans infected by *A. visceralis* in Europe and Australia. The swans examined herein most likely died from obstructive phlebitis associated with *A. visceralis*, but further molecular confirmation is required for identification of this species. However, the present study does not provide new data on the zoonotic potential, but only on the pathogenic potential of this schistosome in swans. Furthermore, our study provides a novel contribution to the description of the pathological effects of avian schistosomes infection in whooper swans in Japan.

## 1. Introduction

Avian schistosomes are a specialized group of parasites and have a particular importance due to their zoonotic potential [1,2,3]. These schistosomes inhabit the circulatory system of the definitive avian hosts and are commonly known as “blood flukes.” Various species of birds can be infected with schistosomes, but infection is most prevalent in waterfowl [1,4,5,6,7]. Among others, *Trichobilharzia* is the largest genus of avian Schistosomatidae, covering more than 40 avian species [8], and they can be divided into visceral and nasal species based on their predilection sites. Visceral species migrate through the viscera and are typically found in mesenteric, renal, cloacal, and portal blood vessels [9,10].

Most of these digenean trematodes have an indirect lifecycle involving a gastropod intermediate host. Fresh-water snails are required for the development of schistosomes belonging to the genera *Bilharziella, Ornithobilharzia, Jilinobilharzia, Macrobilhazia,* and *Trichobilharzia* and salt or brackish mollusks are instead required for genera *Dendritobilharzia*, *Gigantobilharzia,* and *Austrobilharzia* [3,8,10,11]. Infected aquatic bird excrete schistosome eggs, that hatch and release miracidia that penetrate inside the intermediate hosts where they develop to cercaria [11,12]. Thereafter, cercaria is released from the snail intermediate hosts and invade the warm-blooded vertebrate host via skin penetration, in whose circulatory system they develop to adults [1,4,7].

Avian schistosomiasis is characterized by lesions similar to those present in mammals infected with schistosomes, e.g., obliterative endophlebitis, venous hypertrophy, necrosis, granulomatous reaction combined with a mixed inflammatory response, thrombosis of mesenteric veins, fibrinohemorrhagic colitis, and portal fibroplasia, due to the presence of adults and/or eggs in blood vessels [5,9,13,14,15,16,17,18,19,20,21]. However, extensive anatomopathological studies on avian schistosomiasis are lacking. In humans, the repeated penetration of the skin causes the human cercarial dermatitis (HCD), an allergic reaction also known as “swimmer’s itch” [1,15,22]. Cercarial dermatitis can be caused by several species of avian schistosomes, e.g., those belonging to the genera *Trichobilharzia*, *Gigantobilharzia*, and *Austrobilharzia* [3,8,10,11,23]. The zoonotic potential of other species, such as the swan schistosome *Allobilharzia visceralis* remains unknown [6,24,25], despite it cannot be excluded that it can produce HCD. Human cercarial dermatitis is considered endemic in Japan and rice farmers are among the occupational groups at high risk of infection [1,15,26]. In many cases, an etiological diagnosis of HCD at species level can be difficult to achieve, and it cannot be excluded that some cases could be due to *A. visceralis*. Swans are present in Japan and can transport their schistosomes during their migrations [15,23,27]. Indeed, *A. visceralis* has been already reported in swans either in Japan other than in North America and Iceland [13,28,29,30]. Therefore, in the light of the scarce knowledge on the pathology of avian schistosomiasis and of the potential zoonotic implications of blood flukes infecting swans [1,15,31,32], this study evaluated the occurrence of blood flukes in swans from Japan, discussing anatomopathological findings.

## 2. Materials and Methods

### 2.1. Ethical Statement

This study was approved by the Animal Care and Use Committee of Gifu University (approval I: EA07-05) and the Department of Veterinary Pathology, Kafrelsheikh University. Appropriate Institutional Animal Care Guidelines were followed during all handling and procedures.

### 2.2. Animals and Sample Collection

Fifty-four swans were received from rescue/rehabilitation centers in Honshu, Japan, between May 2005 and June 2007. Of these 54 birds, 38 were found dead, 12 swans died shortly after their arrival, whereas 4 swans were severely or moderately emaciated and dehydrated and were euthanized to avoid them unnecessary suffering. To determine the cause of death, the swans were submitted to the veterinary pathology department of the Gifu University for postmortem examination. Thirty-one swans were young (24–36 months), and the remaining were adults (48–72 months). Birds were categorized as immature if they still had brown feathers in their plumage, a pink-gray beak, and gray feet, whereas birds with fully white plumage, an orange beak, and black feet were categorized as mature [19,33]. This study followed the guidelines and measures of the Department of Veterinary Pathology, Gifu University, Japan, for the care and use of animals. In addition, before proceeding with euthanasia, appropriate veterinary care consistent with international, national, and institutional guidelines was guaranteed to the swans.

### 2.3. Gross and Histopathological Examination

Necropsies were performed on all swans in accordance with a standardized protocol. This procedure began with a review of the relevant history followed a full examination to detect anatomopathological alterations. To identify small foreign fragments or any lesions, the contents of the gizzard and intestine were carefully washed. Tissue samples were collected from all organs and from any noticeable lesions. The specimens were fixed in 10% phosphate-buffered formalin, embedded in paraffin, sectioned at a thickness of 4 µm, and then stained with hematoxylin and eosin [34]. Liver sections were also stained with Hall’s bilirubin stain for bile pigment, and specimens of the liver and spleen were stained with Berlin blue stain for iron [34,35].

### 2.4. Parasitological Examination

It is difficult to describe the morphological features of the worms in this study, because the adult worms were very small and typically present only in the lumen of the affected veins. The different stages of the parasites observed in the various histopathological sections were incidentally reported during the measurement of lead (Pb) content in the soft tissues (liver and kidney). The morphology of the different stages of the adult worm in the blood vessels of different damaged internal organs and the effect of eggs in the intestinal mucosa of the affected birds were used for localization of the parasite during histopathological examination, as reported elsewhere [36].

## 3. Results

### 3.1. Gross Examination

Thirty-three out of 54 necropsied whooper swans were severely emaciated and lean, with atrophied pectoral muscles, serious atrophy of pericardial fat, and an absence of fat depots. Twenty-three birds showed multiple reddened areas throughout the entire length of the intestine, which were most prominent in the ileum, cecum, and colon. The main histological lesions were found in the intestinal tract, liver, adrenal gland, spleen, and testis; these lesions were caused by both worms and eggs (Table 1).

### 3.2. Histopathological Examination

Adult schistosomes were found in 43% (23/54) of the swans. In these cases, one or more adult worms were detected in the veins but not in the arteries. Adult worms were found in the lumen of the mesenteric veins, serosal veins, portal veins, testicular veins, capillaries of the intestinal lamina propria, and sinusoids of the adrenal gland, spleen and liver. Macrophages containing brownish pigment were occasionally seen in the parenchyma surrounding the worms, most commonly in the splenic veins and the hepatic sinusoids. Some affected veins, mainly the serosal and mesenteric veins, showed endophlebitis, irregular intimal thickening due to the infiltration of inflammatory cells, and hypertrophy of the muscular fibers in the tunica media.

#### 3.2.1. Intestine

**Figure 1:**
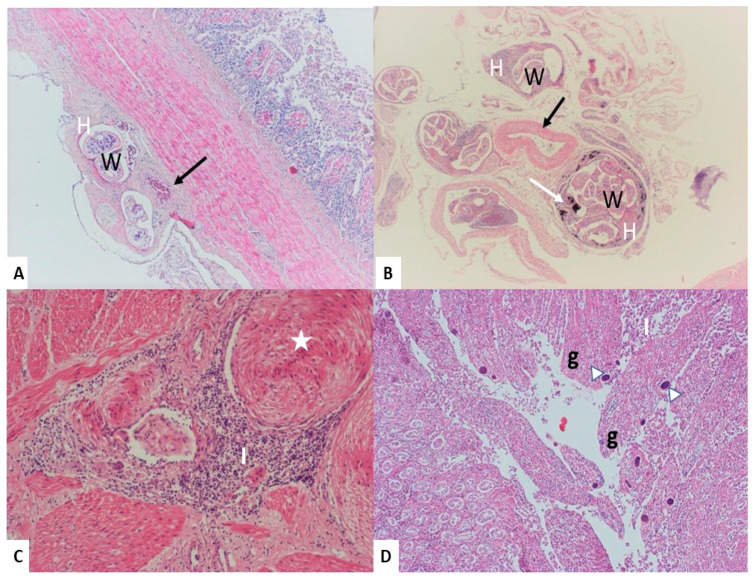
Effect of the parasite in the intestine. (**A**) Adult worms (W) are seen in the thickened walls of the serosal vein (H). Note the normal thickness of the artery (black arrow). (**B**) Brownish pigment–laden macrophages (white arrow) around the worm (W) in the thickened wall of the mesenteric veins (H). (**C**) The vein lumen was almost occluded due to marked myointimal hyperplasia in the veins of the muscular layer of the intestine (white star), with perivascular inflammatory reaction (I). (**D**) Several schistosome eggs (white arrowheads) present in the intestinal lamina propria and surrounded by an inflammatory reaction of lymphocytes and plasma cells (I); the intestinal villi are markedly blunted (g). Hematoxylin and eosin stain (100×).

Numerous sections of adult schistosomes were found in the mesenteric and serosal veins throughout the intestine in all 23 infected birds, being the ileum, caecum, and colon the most affected areas. Vessels containing adult schistosomes showed endophlebitis characterized by myointimal hyperplasia, with infiltration of plasma cells, heterophils, and eosinophils in the surrounding tissues (Figure 1A,B). In 12 swans, the mesenteric and serosal veins, along with the veins between the muscular layers of the intestine, exhibited nodular hypertrophy of the tunica media. Eight swans suffered from obliterative endophlebitis with complete occlusion of the venous lumen (Figure 1C). Superficial enteritis was present in eight birds, and mild to severe villous atrophy was observed in association with numerous parasitic eggs in the lamina propria (in both the small and large intestine) have been observed by microscopical or histological evidence. The eggs were surrounded by venous congestion and diffuse infiltration of the lamina propria, with a variable number of lymphocytes and a few plasma cells, heterophils, and eosinophils (Figure 1D).

#### 3.2.2. Liver

**Figure 2:**
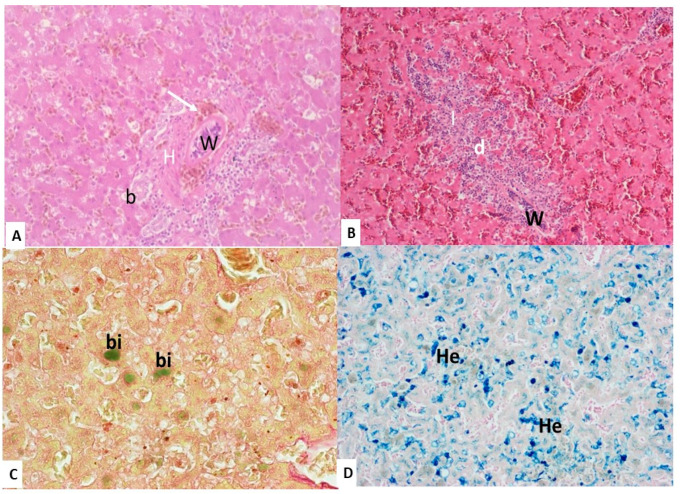
Effect of the parasite in the liver. (**A**) Adult worms (W) present in the thickened walled of the portal vein (H) with hemosiderin pigment deposition (white arrow) and bile duct hyperplasia (b). (**B**) Schistosome parasite (W) found in the hepatic sinusoids surrounded by degenerated hepatocyte (d) and massive inflammatory reaction (I). Hematoxylin and eosin stain (100×). (**C**) Emerald green–colored clumps of bile pigment (bi), Hall stain (400×). (**D**) Macrophages laden with bluish pigment (He), Berlin blue stain (100×).

Adult worms were found in the portal veins and hepatic sinusoids of 14 birds. In five cases, mild to severe inflammatory reactions were observed around the parasites or eggs in the hepatic parenchyma, with degeneration of the hepatic cells and infiltration of a large number of inflammatory cells in the hepatic sinusoids and the portal triads (Figure 2A,B). Fourteen swans had areas of *bile pigment deposition* (biliverdin) in the liver parenchyma, which stained positively with Hall’s stain (Figure 2C). In addition, mild to marked deposition of hemosiderin pigment, positive on Berlin blue staining, was apparent in the liver of 18 swans (Figure 2D).

#### 3.2.3. Adrenal Glands

**Figure 3:**
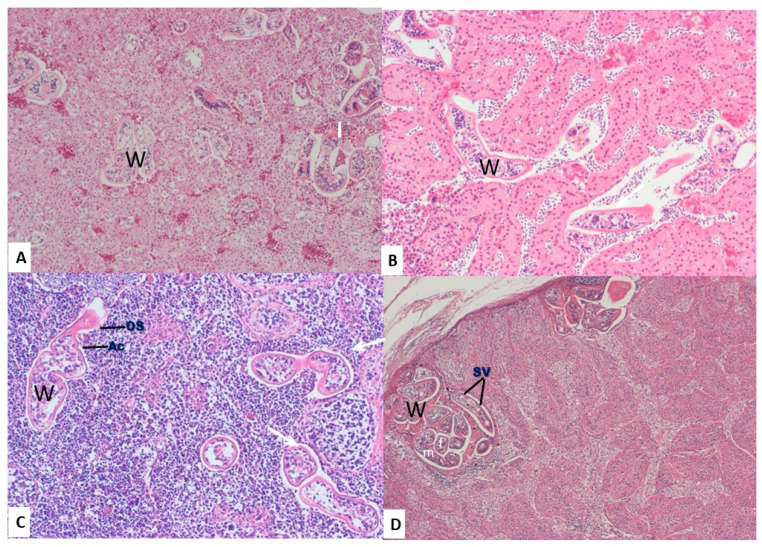
Effect of the parasite in the adrenal gland, spleen, and testis. (**A**, **B**) Multiple cross-sections of adult worms (W) found in the sinusoids of the adrenal gland surrounded by infiltration of mononuclear inflammatory cells (I). (**C**) Adult worms (W) found in the splenic veins. Note the oral sucker (Os) and acetabulum (Ac) of the parasite. (**D**) Multiple cross-sections of adult schistosomes (W) completely occlude the subcapsular veins of the testis and were surrounded by the infiltration of mononuclear inflammatory cells (I). Note the seminal vesicles of the parasite (Sv) and the presence of female (f) in the ventral groove of the male (m) parasite. Hematoxylin and eosin stain (100×).

Numerous sections of adult schistosomes were observed within the adrenal sinusoids of 10 swans, and some sinusoids were completely occluded, resulting in pressure on the surrounding cells (Figure 3A). An inflammatory reaction was seen around the worms in six birds, with infiltration of lymphocytes and plasma cells and degeneration of the adrenal gland cells (Figure 3B).

#### 3.2.4. Spleen

Adult worms were present in the subcapsular sinusoids and splenic veins of 10 swans (Figure 3C). Furthermore, mild to marked deposition of hemosiderin pigment was reported in the spleen of 18 swans (Figure 2D).

#### 3.2.5. Testes

Multiple adult worms were found in the superficial subcapsular veins underneath the tunica albuginea in three swans. In these cases, infiltration of inflammatory cells, predominantly lymphocytes, macrophages, and a few heterophils, was seen in the surrounding parenchyma (Figure 3D).

### 3.3. Parasitological Findings 

Based on the available information, including the species of the affected bird and its location, the morphological characteristics of the schistosome worm and eggs in the intestinal lamina propria, and pathological findings, the schistosomes of the present study were assumed to be *A. visceralis*. The death of the investigated swans was suspected to be due to obstructive phlebitis associated with the suspected parasite. Eggs were found abundantly in the intestinal lamina propria and were mostly ovoid to asymmetrical in shape (Figure 1D); however, confirmation at the species level requires further molecular investigation. Moreover, the schistosome flukes occurred in abundance in the veins of different internal organs with varying morphological characteristics, i.e., oral sucker, posterior sucker, or acetabulum), seminal vesicles, and testis (Figure 3C). Some adult schistosome parasites were found in pairs, with the male carrying the female in its ventral groove or gynecophoric canal (Figure 3D).

## 4. Discussion

Schistosomes are considered to be highly pathogenic in migratory waterfowl [37]. Because the worm is very small and located in the blood vessels, schistosomes in whooper swans can be overlooked on gross necropsy [9,36]. It should be stressed that Swimmer’s itch is considered an emerging disease in various parts of the world, resulting in various nervous or pulmonary symptoms on the basis of the infecting species [38]. Furthermore, avian schistosomes have been considered the most neglected parasitic zoonosis among aquatic birds worldwide [23]. To the best of our knowledge, there are no published studies describing the pathological effects of schistosome infection in whooper swans in Japan. Thus, this study provides a novel contribution to this research topic and adds knowledge on a potentially zoonotic parasite.

We detected adult schistosomes in 43% (23/54) of whooper swans on necropsy; the worms were mostly present in the veins of the large and small intestine, liver, adrenal gland, spleen, and testis. These findings are consistent with previous studies reporting that vascular lesions caused by avian schistosome infection in birds are comparable with lesions observed in visceral schistosomiasis in mammals [9,13,20]. Given the fact that this study was carried out during the period 2005–2007, we planned to perform molecular characterization of the parasite but there were no available samples to do perform this step. In Japan, Hayashi et al. (2017) [13] detected for the first time *A. visceralis* in the capillaries of several organs of whooper swans using molecular methods, without microscopical or histological evidence of eggs or worm. Meanwhile, studies performed in Iceland [28,29] and North America [30] identified vascular lesions caused by schistosomes in whooper swans and tundra swans, respectively. We believe that emaciation and weakness in all 23 infected swans may have resulted from the severe vascular effects associated with schistosome infection, namely, the obstruction of venous return in the mesenteric, intestinal, splenic, and portal veins as well as enteropathy. Both adult schistosomes and eggs contributed to the enteropathy: the adults caused obliterative vascular lesions, whereas the migration of the eggs led to enteritis with villous atrophy [19]. However, in cases such as these, it is not clear whether the parasites are the cause of emaciation [31,39], as important data, such as bodyweight and parasitic load of healthy birds for comparison, are missing [15]. We did not have data on the body weight and parasitic load of apparently healthy whooper swans from this region for comparison. In the current study, the blood flukes infecting the whooper swan were assumed to likely be *A. visceralis*, on the basis on the species of the affected bird, morphological characteristics of the worm and eggs found in the internal organs, and pathological findings associated with their presence in the blood vessels of different organs in both adult and juvenile birds. This method of identification was previously accepted and is in agreement with the findings of Kolářová et al. (2010) [36]. However, further accurate identification methods (i.e., molecular characterization methods are still needed. We were unable to describe the morphological features of adult worms in this study because of the difficulty in obtaining intact specimens: adult worms are very small and are usually present only in the lumen of affected veins, which is consistent with some previous reports [11,19]. Furthermore, these findings are supported by the results from Hayashi et al. (2017) [13], who did not detect egg-like structures of the schistosome in the swans but there was no any gross abnormality or necrosis in the organs of affected swans and assumed that schistosomes did not cause severe damage to the swans examined. Similarly, a recent study reported unidentified schistosomes and their eggs only by histopathological examination [18]. In this study, flukes were found inside the lumen of blood vessels of the muscular layer and in the mucosa of the esophagus, intestine, and caeca but with a slight inflammatory response in only three cases, represented by hemorrhage and infiltration of some inflammatory cells, such as lymphocytes and heterophiles [18]. In contrast, Brant (2007) [24] detected adult worms and egg-tissue debris in a nodule of the inferior mesenteric vein and suggested that, to identify the species of parasite, it is important to investigate the correlation between the pathological lesions and the general condition of the swans, as well as to determine the presence or absence of eggs. The disease features were similar to those described in several reports of schistosome infection in swans from Europe and Australia, including the black swan (*Cygnus atratus*) and the mute swan (*Cygnus olor*) in Australia [27,40] and the whooper swan in Europe [27]. Our results indicated that the number of young birds affected was higher than the numbers of adults, i.e., 61% (14/23) of affected swans were immature. The higher prevalence of infection in the young birds might be due to lower immunity, as compared with the increased immunity in adults that results from repeated exposure to schistosome parasites [41]. In the present study, most infected swans 78% (18/23) showed endophlebitis of the veins containing adult worms, with infiltration of leukocytes into the surrounding tissues. This may have resulted from the irritation of the vessel walls caused by the worms, which led to myointimal hyperplasia and, in some instances, to occlusion of the affected blood vessels. This concept and previous findings are consistent with the reports of Warren (1977) [42], Bolhuis et al. (2004) [19], and Kolářová et al. (2001) [43], all of whom suggested that viable adult schistosomes cause proliferation of the vascular intima either via mechanical injury to the intima or via the secretion of antigens as an immune (allergic) reaction against the schistosomes. Bolhuis et al. (2004) [19] revealed the occurrence of *Trichobilharzia* sp. in five of eight mute swans, whereas pathological lesions including moderate to severe, diffuse, hyperplastic endophlebitis were reported in the intestinal veins together with splenic and hepatic hemosiderosis [19]. In the current study, we did not find any fibrosis in the portal triad in the birds examined. This is consistent with the findings of Kolářová et al. (2006) [15] but in contrast to those of Bolhuis et al. (2004) [19], who reported mild to extensive fibroplasia of the portal triads in mute swans and attributed this finding to the aberrant localization of female schistosomes in the bile ducts. Similarly, Wojcinski et al. (1987) [44] reported the same lesions in ducks infected with schistosomes but found no evidence of parasites in bile ducts, and Robinson and Maxie (1993) [20] reported that in mammals, severe cases of schistosomiasis might result in occlusion of the lumen of the veins, and lesions may extend to the intrahepatic branches of the portal vein, leading to prominent portal fibrosis. Bolhuis et al. (2004) [19] also reported the development of granulomas due to the severe perivascular inflammatory reaction around the veins harboring the adult parasite in the liver of juvenile and adult birds; in contrast, Brant (2007) [24] did not find any granulomas in the portal triads of infected swans.

In the present work, worm eggs were found multifocally in the lamina propria of the small and/or large intestine, and 35% (8/23) of swans showed mild to severe villous atrophy (villous blunting, fusion, and edema) and superficial enteritis, which might have resulted from the migration of schistosome eggs through the intestinal wall. This explanation is supported by a previous study conducted by Wojcinski et al. (1987) [44]. In addition, in their study, Bolhuis et al. (2004) [19] reported lymphocytic and granulocytic enteritis associated with the presence of eggs of schistosomes in the intestinal mucosa of examined swans. However, Horák et al. (2002) reported that in human schistosomiasis, some eggs fail to reach the intestinal lumen and are instead disseminated through the circulatory system to the liver, lungs, and other organs, where they cause reactions ranging from very mild to marked granulomatous inflammation. Robinson and Maxie (1993) [20] stated that schistosome eggs release antigens that induce a delayed hypersensitivity response and cause the formation of small granulomas, which are characterized by the infiltration of eosinophils, mononuclear leukocytes, and giant cells as well as reactive fibrosis. In the present study, we observed bile pigments and hemosiderin in the liver of 61% (14/23) and 78% (18/23) of whooper swans infected with avian schistosomes, respectively, whereas adult worms and/or eggs were detected in the liver of 61% (14/23) of infected swans. This indicates that cholestatic jaundice may be caused by avian schistosomiasis, as found by Akagami et al. (2010) [16]. Schistosomes were detected in the sinusoids of the adrenal gland in 44% (10/23) of swans in the present study; this finding has not previously been reported, except in one study by Hayashi et al. (2017) [13]. A unique finding of this study is the detection of worms in the sinusoids of the testes in 13% (3/23) of infected swans. We believe that the presence of parasites in the adrenal glands and in the testes was due to the migration of parasites through the vascular system of heavily infected birds.

## 5. Conclusions

In conclusion, our study provides interesting data that describe the pathological effects of schistosome infection, assumed to be *A. visceralis*, in whooper swans in Japan, which provides a novel contribution to this field of research. It could be suggested that these parasites were the cause of death in the infected swans in this study, because they were found in the blood vessels of all infected swans and caused obstructive phlebitis. Additional molecular studies on schistosome infection in whooper swans and in their snail intermediate hosts are required to better characterize the parasite species.

## Figures and Tables

**Table 1:** Summary of histopathological lesions and worm findings in 23 infected whooper swans.

No. of Swans	1	2	3	4	5	6	7	8	9	10	11	12	13	14	15	16	17	18	19	20	21	22	23
Sex/Age	F/A	F/A	F/A	M/Y	M/Y	M/Y	M/Y	F/A	F/A	F/Y	M/A	M/Y	M/Y	F/A	M/Y	F/Y	ND/Y	F/A	F/Y	F/ND	M/Y	F/A	M/Y
Emaciation	++	+++	+	+	+	++	+	+++	+	+	++	+	+	+	++	+	+	++	+++	++	+	+	+
Parasites in serosal vein	+	++	+	++	+	++	+	++	+	+	++	+	+	++	+	+	+	++	+	+	+	++	++
Parasites in mesenteric V	++	++	+	+	++	++	++	+	+	+	+	+	++	+	+	+	+	+	+	+	+	+	+
Parasites in portal vein	+	+	+	-	-	+	+	++	-	-	+	-	-	-	+	-	+	+	+	+	-	+	+
Parasites in hepatic sinusoids	+	+	-	+	-	+	+	+	-	-	++	-	-	-	++	-	+	+	+	+	-	+	+
*Parasites in adrenal sinusoids*	+	+++	+	-	-	-	++	-	-	+	-	-	+	-	-	+	-	++	+++	-	-	++	-
Parasites *in splenic veins*	-	-	+	+	-	-	+	+	+++	-	+	+	-	-	-	-	+	+	-	-	+	-	-
Parasites *in subcapsular veins*	+++	++	-	-	-	-	-	++	-	-	-	-	-	-	-	-	-	-	-	-	-	-	-
*Bile pigment deposition*	++	++	-	+	-	+++	-	+++	+	-	++	-	+	-	++	-	-	+	+	+	-	+	+
*Hemosiderin pigment*	+++	++	-	++	+	++	-	+++	++	-	++	-	+	-	++	+	+	++	++	+	+	+	+
Venous hypertrophy	++	++	-	-	++	-	++	++	-	-	++	-	+	-	++	-	+	++	++	-	-	++	-
Obliterative endophlebitis	+	+	-	-	+	-	-	+	-	-	+	-	-	-	++	-	-	++	+	-	-	-	-
Villous atrophy		+	-	-	+	+	-	+	++	-	+	-	-	+	-	-	-	++	-	-	-	-	-

F: female M: male A: adult Y: young NA: not analyzed ND: not determined N: negative + mild ++ moderate +++ severe.
